# Diagnosis, evaluation and management of osteoporosis in chronic kidney disease: navigating treatment approaches – Indian consensus statement

**DOI:** 10.3389/fneph.2025.1601610

**Published:** 2025-07-10

**Authors:** Vijay Kher, Rajkumar Sharma, Georgi Abraham, Bharat Shah, Sishir Gang, Sanjeev Gulati, Manisha Sahay, Jatin Kothari, Anil Kumar BT, Raja Ramachandran, Sanjay Kalra, Rakesh Kumar Sahay, Om Lakhani, Jay Kumar Sharma, Deepak Bunger, Thomas L. Nickolas

**Affiliations:** ^1^ Department of Nephrology and Kidney Transplantation, Epitome Kidney Urology Institute and Lions Hospital, New Delhi, Delhi, India; ^2^ Department of Nephrology and Renal Care, Medanta Hospital, Lucknow, Uttar Pradesh, India; ^3^ Department of Nephrology, MGM Healthcare and Hospital, Chennai, Tamil Nadu, India; ^4^ Institute of Renal Sciences, Gleneagles Hospital, Mumbai, Maharashtra, India; ^5^ Department of Nephrology, Muljibhai Patel Urological Hospital (MPUH), Nadiad, Gujarat, India; ^6^ Department of Nephrology and Kidney Transplantation, Fortis Escorts Hospital, New Delhi, Delhi, India; ^7^ Department of Nephrology, Osmania Medical College and Hospital, Hyderabad, Telangana, India; ^8^ Department of Nephrology and Kidney Transplantation, Nanavati Max Super Speciality Hospital, Mumbai, Maharashtra, India; ^9^ Department of Nephrology, Gleneagles BGS Hospital, Bengaluru, Karnataka, India; ^10^ Department of Nephrology, Post Graduate Institute of Medical Education and Research (PGIMER), Chandigarh, India; ^11^ Department of Endocrinology, Bharti Hospital, Karnal, Haryana, India; ^12^ Department of Endocrinology, Osmania Medical College and Hospital, Hyderabad, Telangana, India; ^13^ Department of Endocrinology, Zydus Hospital, Ahmedabad, Gujarat, India; ^14^ Department of Medical Affairs, Intas Pharmaceuticals Limited (Corporate Office), Ahmedabad, Gujarat, India; ^15^ Department of Medicine, Division of Nephrology, Columbia University Irving Medical Center, New York, NY, United States

**Keywords:** osteoporosis, chronic kidney disease, expert consensus, renal osteodystrophy, mineral and bone disorder, bone turnover markers, bone mineral density

## Abstract

**Background:**

Managing osteoporosis (OP) in chronic kidney disease (CKD) presents significant challenges due to altered bone metabolism. Given the lack of robust clinical trial data and a notable knowledge gap exists among nephrologists regarding an optimal management in this population, an expert consensus is crucial for developing tailored management strategies. This study aimed to gather an expert opinion to bridge this gap and establish consensus recommendations on the diagnosis and management of osteoporosis in CKD patients.

**Methods:**

A panel of 13 Indian and 1 international experts, including nephrologists and endocrinologists, participated in a structured survey and discussion process. Thirteen Indian experts provided their opinion on key clinical issues, including screening, diagnosis, and treatment strategies for osteoporosis in CKD. Consensus was achieved in a single round of voting, and recommendations were formulated based on the level of agreement among the panelists.

**Results:**

The expert panel reached a strong consensus (80-100% agreement) on several critical recommendations. It was agreed that osteoporosis in CKD is often asymptomatic, with fragility fractures being less common, and thus, early screening using dual-energy X-ray absorptiometry (DXA) is essential. The panel emphasized the importance of evaluating bone turnover status using serum biomarkers such as bone-specific alkaline phosphatase (BSAP) and intact parathyroid hormone (iPTH) to guide treatment decisions. Tailored treatment strategies were recommended, with a judicious use of bisphosphonates and denosumab, depending on the patient’s estimated glomerular filtration rate (eGFR) and bone turnover state. The management of renal osteodystrophy (ROD) was deemed necessary before addressing CKD-induced osteoporosis.

**Conclusion:**

This expert consensus provides critical insights and guidance for the management of osteoporosis in CKD. The recommendations emphasize individualized treatment approaches, the importance of early screening, and the integration of multidisciplinary care. These findings aim to fill existing knowledge gaps and improve clinical outcomes for CKD patients with osteoporosis.

## Graphical Abstract

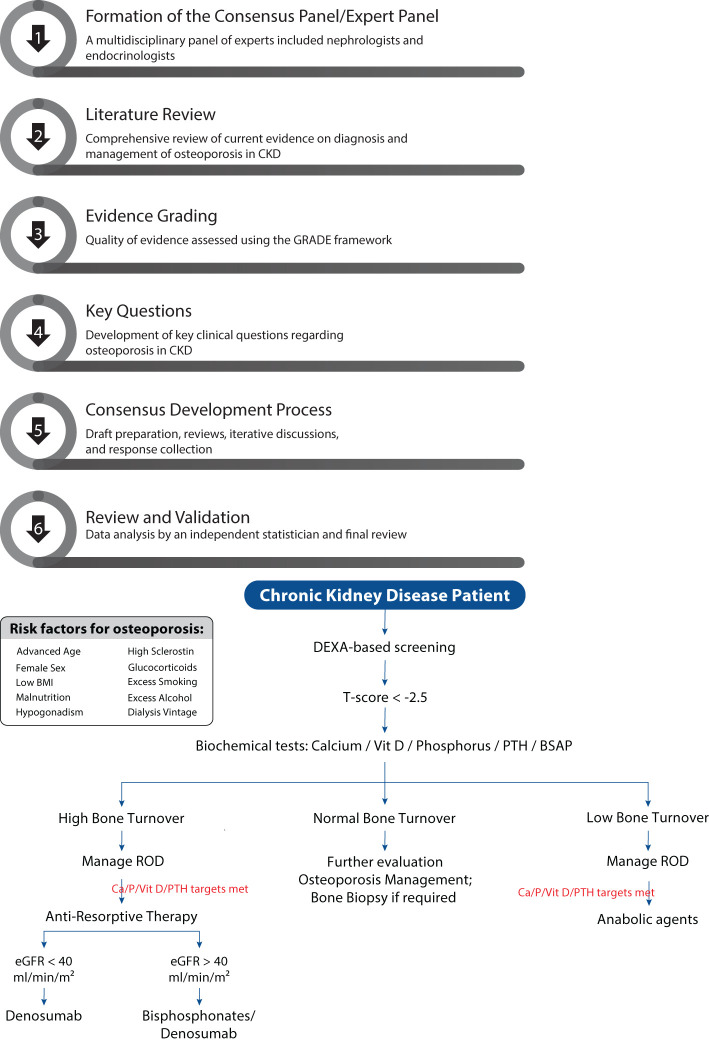

## 1 Introduction

Osteoporosis is a major public health concern, affecting millions globally as well as India leading to a significant morbidity, mortality, and healthcare costs. In India, the prevalence of osteoporosis among Chronic Kidney Disease (CKD) patients on hemodialysis is notably high, with studies showing prevalence of approximately 17 – 31.8%. The burden of osteoporosis in these patients is compounded by CKD-related mineral and bone disorder (CKD-MBD), increasing their risk for fragility fractures and further exacerbating morbidity and healthcare costs. In the general population, osteoporosis typically arises due to age-related bone loss and postmenopausal changes (1, 2). The pathophysiology is more complex and multifactorial, involving CKD-MBD, secondary hyperparathyroidism, altered bone remodeling dynamics, metabolic disturbances and chronic inflammation (3).

CKD is associated with disturbances in calcium, phosphorus, vitamin D, and parathyroid hormone metabolism. These disturbances contribute to abnormalities in bone turnover, mineralization, volume, linear growth, or strength, collectively recognized as CKD-MBD. Patients with CKD are at an increased risk for fractures due to these alterations in bone metabolism, coupled with the direct effects of uremia on bone and muscle function (3, 4). The diagnosis and management of osteoporosis in CKD patients are challenging due to the overlapping features of CKD-MBD and primary osteoporosis, which further complicate the clinical landscape. Traditional diagnostic tools such as dual-energy X-ray absorptiometry (DXA) scans, which measure bone mineral density (BMD), may not fully capture the bone quality in CKD patients. Factors such as vascular calcification and altered bone composition necessitate a re-evaluation of BMD results in this population. Additionally, biomarkers of bone turnover and advanced imaging techniques, such as high-resolution peripheral quantitative computed tomography (HR-pQCT), may offer more insight into the underlying bone pathology in CKD patients (5).

Management of osteoporosis in CKD involves a multifaceted approach, including lifestyle modifications, pharmacologic therapies, and addressing the underlying CKD-MBD problem. Pharmacologic options are often limited by the stage of CKD and the overall health status of patients. For example, bisphosphonates, commonly used in the general population, pose risks of adynamic bone and renal toxicity in advanced CKD (6). Newer agents, such as receptor activator of nuclear factor-kB ligand (RANKL) inhibitors, selective estrogen receptor modulators (SERMs), and anabolic medications, such as sclerostin inhibitors, type 1 parathyroid hormone receptor (PTH1R) ligands, offer alternative therapeutic options but require careful consideration in a view of their renal implications and potential side effects (7). Given the complexities involved and the knowledge gap existing among nephrologists regarding management of osteoporosis in the Indian population, a consensus on the optimal approach to diagnose and manage osteoporosis in CKD is essential. Therefore, the objective of this expert consensus was to bridge this gap and provide an overview of current best practices for the diagnosis and management of osteoporosis in CKD. This consensus statement integrates current guidelines, recent research findings, and experts’ opinion to address the unique needs of this patient population, emphasizing the importance of early identification, accurate diagnosis, and individualized treatment strategies. By establishing a standardized approach, we hope to improve patient outcomes, reduce fracture risk, and enhance quality of life for those affected by both osteoporosis and CKD-MBD.

## 2 Methodology

### 2.1 Formation of the consensus panel/expert panel

A multidisciplinary panel of experts was formed to develop the consensus statement on the diagnosis and management of osteoporosis in CKD. The panel included nephrologists and endocrinologists with vast experience in managing patients with osteoporosis and CKD-MBD. The selection of panel members was based on their clinical expertise, academic contributions, and involvement in relevant professional societies.

### 2.2 Literature review

A comprehensive literature review was conducted to gather current evidence on the diagnosis and management of osteoporosis in CKD. The search included peer-reviewed articles, clinical trials, systematic reviews, meta-analyses, and existing guidelines published in English from 2000 to 2023. Databases such as PubMed, MEDLINE, Embase, Cochrane database Library, Web of Science and Google Scholar were used to identify relevant studies. Keywords included “osteoporosis,” “chronic kidney disease,” “CKD-MBD,” “bone mineral density,” “fractures,” “diagnosis,” “management,” and “treatment.” The quality of evidence was evaluated to provide a robust foundation for developing the questionnaire on osteoporosis management in CKD patients. For each clinical question, the level of evidence was determined to provide a basis for developing the consensus statement or recommendation.

### 2.3 Evidence grading

The quality of evidence was assessed using the Grading of Recommendations, Assessment, Development, and Evaluation (GRADE) framework (8). Each study was evaluated based on its design, sample size, methodology, consistency of results and relevance to clinical practice. This grading system provided a systematic approach to determine the confidence in effect estimates for each clinical question addressed. The level of evidence for each recommendation was determined, ensuring that the consensus statement was grounded in the most reliable and relevant data available. This rigorous assessment guided the development of robust, evidence-based guidelines.

### 2.4 Key questions used to develop the consensus statement

To guide the development of the consensus statement, the expert panel formulated a total of 11 key clinical questions addressing critical aspects of osteoporosis in CKD. These questions encompassed a wide range of topics to ensure comprehensive coverage of all available information. They focused on the prevalence and risk factors of osteoporosis in CKD patients, identification of optimal diagnostic methods, including the role of bone mineral density (BMD) assessments and advanced imaging techniques, and differentiation between osteoporosis and CKD-related bone disorders. Additionally, questions were framed to address effective pharmacologic and non-pharmacologic treatment strategies tailored to different CKD stages and the management of fracture risk in this population. The evidence to answer the clinical questions was collected in the form of agree, disagree, and neutral types of options. These questions, shown in **Table 1**, form the basis of the systematic literature search and consequently the clinical care standards and practice. These questions were drafted to ensure comprehensive coverage of relevant topics and were used to direct the literature review, evidence grading, and consensus development processes, resulting in well-rounded, evidence-based recommendations.

**Table 1:** Final recommendations on diagnosis and management of osteoporosis in CKD.

Sr No	Final statement or recommendations	Consensus	Panelist comments
**1**	Real-world data reveal a prevalence of osteoporosis in 17-20% of dialysis-dependent CKD patients Your take on this data.	84.6%	This data is under represented and the actual prevalence is much higher. Therefore the problem requires immediate attention.
**2**	The most common presentation of CKD induced osteoporosis is asymptomatic (detected during screening) followed by fragility fractures.	92.3%	The most common presentation of CKD induced osteoporosis is diagnosed on screening, therefore CKD patients must be screened periodically for early detection of osteoporosis
**3**	Osteoporotic CKD patients having T-score ≤ -2.5 or those with normal BMD and history of fragility fracture require treatment for osteoporosis.	100%	Even if BMD normal, history of fragility fracture is an indication for osteoporosis treatment
**4**	Multidisciplinary approach is required for management of CKD- induced osteoporosis	100%	Cross referral can be done whenever necessary. Nephrologists must screen the patients who presents with risk factors for Osteoporosis.
**5**	Renal osteodystrophy (ROD) management is necessary prior to managing CKD induced osteoporosis	92.3%	The panel concurs that managing ROD is essential before addressing CKD-induced osteoporosis. Effective ROD management helps stabilize bone metabolism, ensuring that subsequent osteoporosis treatments are more effective and tailored to the patient’s needs. Biochemical parameters like Calcium, Phosphate, Vitamin D and PTH can also be utilized. Bone biomarkers can be used to monitor that the patient is compliant to the treatment or not and to assess the effectiveness of treatment.
**6**	Follow up of osteoporotic patients should be done by evaluation of BMD changes using DXA	92.3%	Follow up of the patients of CKD-associated osteoporosis can be done either yearly or two yearly, evaluating BMD changes. The panel agrees that follow-up of osteoporotic patients should include regular evaluation of BMD changes using DXA scans. This method provides a reliable measure of bone density and helps in monitoring the effectiveness of treatment interventions over time.
**7**	Wherever available, bone-specific alkaline phosphatase (BSAP) is a better marker than intact parathyroid hormone (iPTH) to distinguish high *vs* low bone turnover disease	84.6%	PTH is usually considered to screen the patients for high bone turnover. As BSAP is not cleared by kidneys, it can be a more suitable marker. Combining both BSAP and PTH can be more helpful in differentiating High *vs* low bone turnover disease.
**8**	DXA is the investigation of choice for screening of osteoporosis.	100%	Non-DXA radiology (quantitative computed tomography, vertebral fracture assessment etc.) is useful in only symptomatic patients. The panelists recommended incorporating Trabecular Bone Score (TBS) alongside DXA to enhance the assessment of bone microarchitecture, wherever available. However, TBS seems to be effective in pre-hemodialysis (HD) patients only, as there is limited data available for its use in HD patients.
**9**	The management of CKD associated osteoporosis starts with life style management, ROD management and specific therapies as tested by serum BSAP. High bone turnover can be managed by either Bisphosphonates/Denosumab for eGFR >40 ml/1.73m^2^/min and Denosumab for lesser than this while low bone turnover disease requires anabolic agents	100%	Life style modification including aerobic/weight bearing exercises are recommended. Patient should be put on Calcium and vitamin D supplementation prior to denosumab injection. Calcium levels should be measured more frequently in first 2-to-3 weeks post denosumab injection as chances of hypocalcemia is comparatively higher during this phase.
**10**	The Fracture Risk Assessment Tool (FRAX) is an important tool which can be used in clinical practice for assessing fracture risk.	92.3%	The panel agrees that FRAX is a valuable tool in clinical practice for assessing fracture risk in CKD population with age ≥40 years and should be utilized prior to initiate the treatment. The panel also agreed that FRAX has demonstrated utility in CKD patients across all stages of kidney disease, even without BMD input. This highlights FRAX as a valuable tool, especially in regions where DXA scans are either unavailable or unaffordable. Moreover, use of peripheral Calcaneal quantitative ultrasound (QUS) as a diagnostic tool for osteoporosis is not recommended in patients with CKD, however as a screening method, it can be useful.
**11**	Based on above discussion points, can we formulate the algorithm mentioned below?	100%	The panel believes that, it is feasible to formulate a comprehensive algorithm for the diagnosis and management of osteoporosis in CKD. This algorithm would integrate key steps, including initial assessment, management of ROD and regular BMD monitoring with DXA.

### 2.5 Consensus development process

The consensus development process was conducted through several systematic stages to ensure a rigorous and scientifically sound approach: **Stage A (Initial Draft Preparation):** A core writing group within the expert panel prepared an initial draft of the consensus statement. This draft summarized the key findings from the comprehensive literature review and integrated the expert opinions of the panel members. The goal was to create a foundational document that addressed the critical aspects of osteoporosis management in CKD. **Stage B (Draft Questionnaire Development and Initial Review):** Following the initial draft preparation, a meeting of the panel members was held to review and discuss the draft questionnaire. During this meeting, the panel members suggested modifications and refinements to ensure the questionnaire was comprehensive and accurately reflected the key clinical questions and issues. **Stage C (Iterative Review and Virtual Meeting):** Regular virtual meetings were subsequently held to discuss the questionnaire in detail. These meetings provided a platform for open dialogue and collaborative decision-making among the experts. Disagreements were addressed, and questionnaire was refined based on ongoing discussions. This iterative process ensured that all panel members had the opportunity to contribute their insights and expertise. **Stage D (Final Consensus and Response Collection):** A virtual advisory board meeting was conducted and the final consensus was reached through a systematic process of collecting responses from panel members for each question. Responses were anonymized to reduce bias and encourage honest and objective feedback. This anonymization process helped ensure the integrity of the responses. Areas of significant disagreement were revisited and resolved through further discussion and additional evidence review. The goal was to achieve a high level of agreement (e.g., 80% or higher) for each recommended question, ensuring robust and reliable consensus-based suggestions for the diagnosis and management of osteoporosis in CKD.

### 2.6 Review and validation

**Figure 1:**
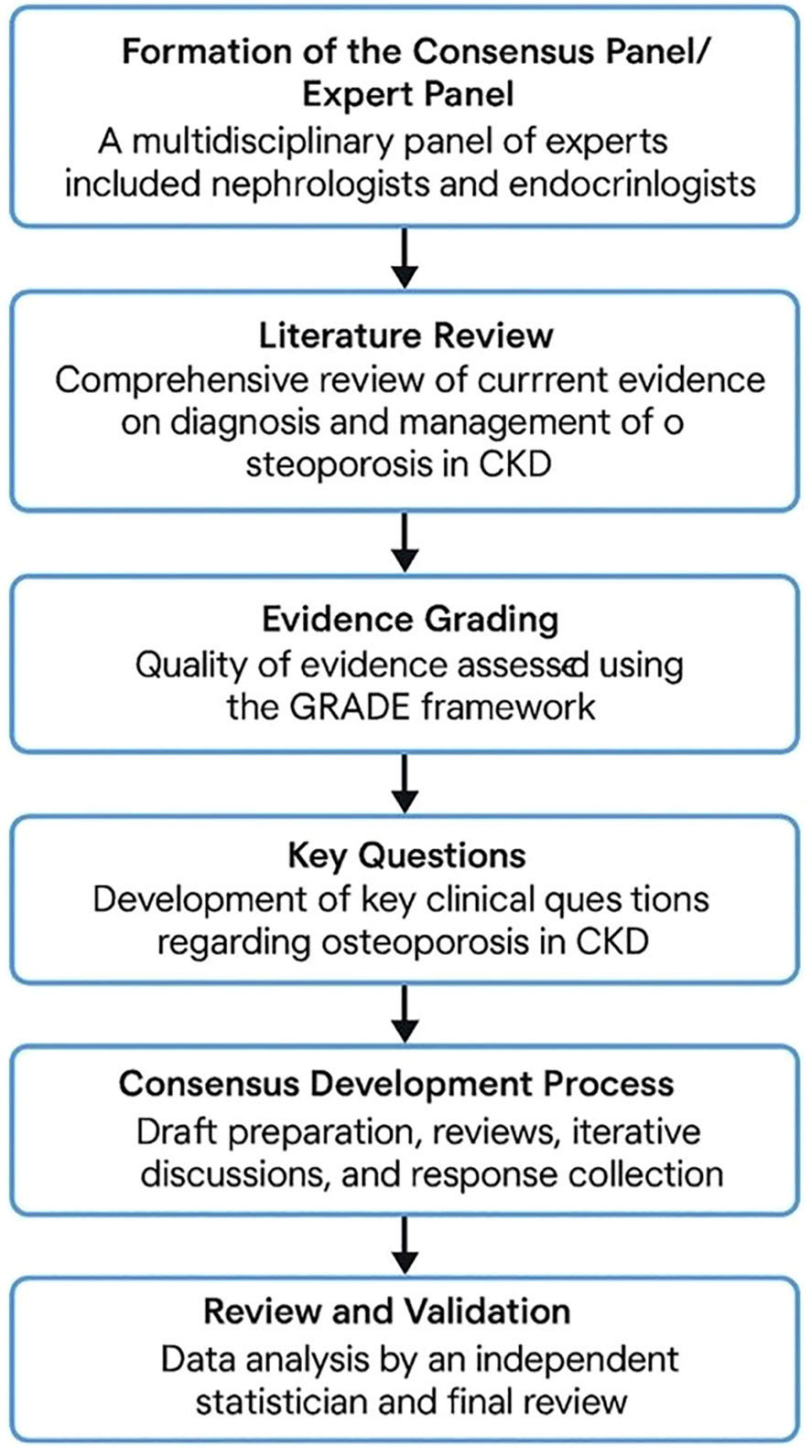
Flow diagram of the consensus process.

The draft consensus statement was subjected to data analysis by independent statistician not involved in the initial panel. Data analysis was incorporated into the final document to enhance its validity and applicability. Additionally, a summary was drafted, reviewed for clarity, consistency, and adherence to methodological standards. The flow diagram of the consensus process is provided in **Figure 1**. The study protocol adhered to the guidelines of the Equator Network and registered in public repository namely, International Prospective Register of Systematic Reviews (PROSPERO with registration number 1056111) to ensure transparency and accuracy in reporting the results.

## 3 Results

### 3.1 Data collection and evidence evaluation

The literature research and evidence selection process was comprehensive and methodologically rigorous, ensuring the inclusion of high-quality and relevant studies. A systematic search was conducted across multiple databases, including PubMed, Embase, Cochrane Library, and Clinical trial (NLM database), covering the period from 2000 to 2023. From the initial search, a total of 132 potential articles were identified. After removing duplicates and screening titles and abstracts, 61 articles were deemed potentially relevant. These articles underwent full-text review by two independent reviewers, resulting in the inclusion of 24 studies that met the predefined criteria (population, intervention of interest, study design or report outcomes and measures) for quality and relevance. Data abstraction and extraction were performed meticulously, focusing on study design, intervention, sample size, methodology, outcomes, and relevance to the key clinical questions. The quality of evidence was assessed using the Grading of Recommendations, Assessment, Development, and Evaluation (GRADE) framework, categorizing the evidence into high, moderate, low, and very low levels (8). Each selected study contributed to answering the 11 key clinical questions formulated by the expert panel. The evidence was used to development of the consensus recommendations, ensuring that they were grounded in robust and reliable data. This rigorous literature research and evidence selection process provided a strong foundation for the consensus statement, enhancing its credibility and applicability in clinical practice.

### 3.2 Expert panel characteristics

The expert panel comprised 14 members, including 13 Indian experts and 1 international expert. Dr. Thomas Nickolas, the international expert and an eminent nephrologist from Columbia University, USA, provided valuable insights into CKD-associated osteoporosis, highlighting the challenges faced in the USA and actively participated in the discussion and debate sessions for questionnaire development, though did not partake in the final expert panel survey. The Indian panel consisted of 10 nephrologists and 3 endocrinologists, all of whom are deeply involved in both the development of the questionnaire draft. All the nephrologists were specialists in management of osteoporosis and bone metabolism disorders associated with CKD, ensuring a comprehensive, multidisciplinary approach to the consensus process.

### 3.3 Development of panel questionnaire

The development of the panel questionnaire was an integral part of formulating the consensus statement, designed to address the multifaceted nature of osteoporosis in CKD comprehensively. Drawing from an extensive literature review and evidence evaluation, we formulated 11 key questions (**Table 1**). These questions spanned several critical domains which include epidemiology, clinical presentation, diagnostic techniques, treatment approaches, and algorithm development. They aimed to capture the prevalence, risk factors, unique symptoms, diagnostic challenges, and multidisciplinary treatment strategies, including both pharmacologic and non-pharmacologic interventions along with formulating the algorithm for osteoporosis in CKD. The final questionnaire employed a structured response format (agree, disagree, neutral, somewhat) to systematically collect expert opinions (**Table 1**).

### 3.4 Expert panel survey

The expert panel survey was completed in a single round via ‘virtual advisory board’, diverging from the typical Delphi consensus approach. This streamlined process involved the collection of responses and comments from the panel members, ensuring a robust and comprehensive consensus. The survey resulted in 11 key recommendations, addressing critical aspects/domains of osteoporosis management in CKD (**Table 1**).

**Figure 2:**
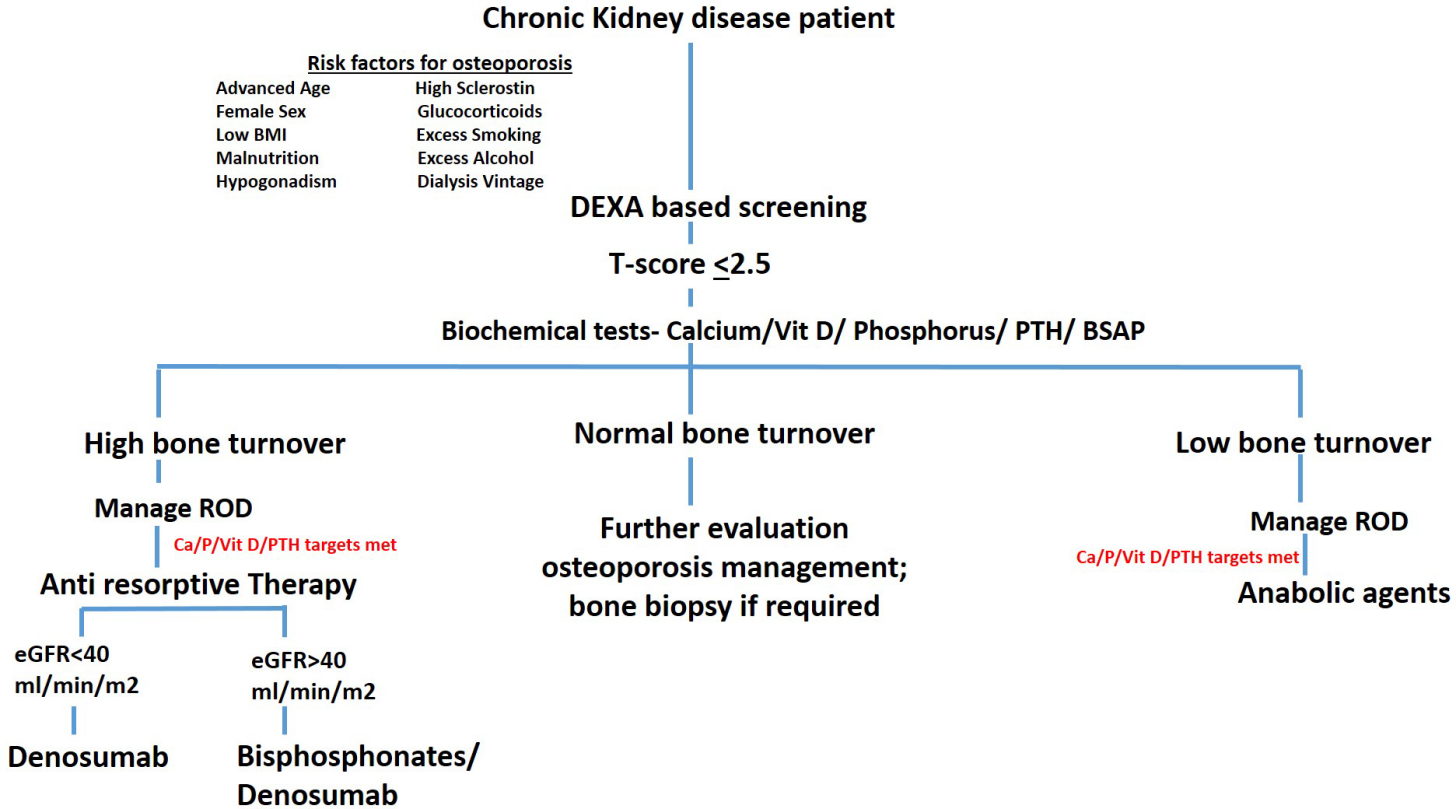
Comprehensive algorithm for managing osteoporosis in CKD. This figure illustrates a step-by-step algorithm for the diagnosis and management of osteoporosis in patients with CKD. The algorithm integrates key diagnostic and therapeutic strategies, starting with initial risk assessment, bone turnover evaluation, and ROD management. It includes BMD monitoring via DXA, and the use of both anti-resorptive and anabolic therapies tailored according to bone turnover status.

The expert panel also supported the use of the FRAX tool as a valuable method for predicting fracture risk in CKD patients. Detailed consensus findings and panelist comments are provided in **Table 1**. Finally, the panel proposed a comprehensive algorithm for managing osteoporosis in CKD, tailored to the specific needs of this population. The proposed algorithmic flow chart is presented in **Figure 2**. The risk factors agreed upon for osteoporosis in CKD included female sex, hyperphosphatemia, hyperparathyroidism, sclerostin overproduction, glucocorticoids usage, hyperprolactinemia, hypogonadism, and malnutrition. Panelists also suggest that malnutrition and hypogonadism are very important issues to address in the dialysis population.

The panel emphasized the utility of BSAP over iPTH in distinguishing high *vs*. low bone turnover. However, 84.6% of panelists suggested that combining both, BSAP and iPTH, could be more beneficial in differentiating High *vs* low bone turnover. Additionally, other bone turnover markers are also classified in two categories based on the metabolic phase during which they produce. The detailed list of biochemical markers are provided in **Table 2**.

**Table 2:** Biochemical markers of bone turnover.

Biomarkers of bone formation	Biomarkers of bone resorption
Osteocalcin	Pyridinolines
Bone specific alkaline phosphatase	Deoxypyridinolines
C-terminal propeptide of type I collagen	Pyridinium crosslinks
N-terminal propeptide of type I collagen	Crosslinking telopeptides of type I collagen (C-terminal, CTx and N-terminal, NTx)
	Tartrate resistant acid phosphatase
	Galactosyl hydroxylysine
	Hydroxyproline

## 4 Discussion

Osteoporosis in chronic kidney disease (CKD) is a complex condition that arises from the intricate interplay between CKD-related mineral and bone disorders (CKD-MBD) and traditional risk factors for osteoporosis. While specific global prevalence rates for CKD-MBD are not uniformly reported, studies indicate that mineral and bone disorders are common among CKD patients, particularly as kidney function declines (9, 10). In the India, CKD-MBD appears to be more prevalent and severe compared to Western populations (11). Few studies conducted in India found a high prevalence of CKD-MBD among CKD patients on hemodialysis, with studies showing that approximately range of 17 - 31.8% of these individuals are affected in India (1, 2). This high prevalence is attributed to factors such as widespread vitamin D deficiency, dietary habits, and limited access to healthcare resources and thereby, linked to the compounded effects of CKD-MBD, which further accelerates bone loss and heightens fracture risk, contributing to significant clinical challenges in managing bone health (1, 2, 11). Moreover, increased CKD-related osteoporosis in developing nations like India is a complex issue with several contributing factors, including limited access to healthcare, economic disparities, nutritional deficiencies, and the prevalence of underlying conditions like diabetes and hypertension. These factors exacerbate the disruption of mineral and bone metabolism that occurs naturally with CKD, leading to weakened bones and increased fracture risk (2). These findings emphasize the importance of early detection and management of CKD-MBD, particularly in regions with higher prevalence rates like India. Additionally, while this consensus statement is primarily informed by Indian experts, it acknowledges that regional differences, such as healthcare infrastructure, disease burden, and access to diagnostic or therapeutic modalities, can influence the implementation of clinical recommendations. The generalizability of these findings to other countries or populations should therefore be considered in the context of local resources and practice environments. This aligns with observations by Kim, 2014 (12) who noted that differences in medical care and social factors between countries can limit the generalizability of global CKD-MBD guidelines. Nevertheless, the core principles align with broader international perspectives and are complementary to regional frameworks like the KDIGO 2017 CKD-MBD guideline updates with Indian commentary (Valson et al., 2020). These efforts reflect a growing need for region-specific adaptations of global guidelines, highlighting the relevance and necessity of such consensus statements tailored to local practice patterns. Patients with CKD are particularly vulnerable to bone loss due to altered calcium and phosphate metabolism, secondary hyperparathyroidism, and vitamin D deficiency. These factors contribute to reduced bone mineral density (BMD) and an increased risk of fractures (3, 4). The diagnosis and management of osteoporosis in CKD require an approach that considers the cross-talk between bone and mineral disorders specific to this population. Managing osteoporosis in CKD is uniquely challenging because standard osteoporosis treatments may not be effective or appropriate in this patient population (13). Therefore, a customized approach that addresses both CKD-related issues and bone health is crucial for improving patient outcomes. The experts in the field reached a consensus on many aspects, including diagnostic challenges and management strategies for patients with osteoporosis in CKD (14). The outcomes of this consensus survey would help to bridge current knowledge gaps and inconsistencies in the current management strategies for osteoporosis in CKD.

Given the high prevalence of this condition and its significant impact on morbidity and mortality, there is an urgent need for evidence-based, expert-driven recommendations. Our expert consensus aimed to address these complexities by developing evidence-based directives and a consensus statement that can guide clinical practice for diagnosing primarily during routine screening and managing the asymptomatic nature of this disease. The recommendations were derived from a systematic evaluation of the literature, coupled with expert opinion, ensuring that the guidelines are both comprehensive and relevant to current clinical challenges. Herein, the expert panel emphasized the need for early and accurate diagnosis, with a multidisciplinary approach to management that includes both pharmacologic and non-pharmacologic interventions (life-style management). By prioritizing the management of renal osteodystrophy (ROD), the recommendations aim to improve patient outcomes through more precise and personalized treatment strategies. The complexity of osteoporosis in CKD requires input from multiple disciplines to ensure that all relevant aspects are addressed (14, 15). By involving experts from nephrology and endocrinology, we were able to incorporate a wide range of perspectives into the discussion and debate.

In expert opinion surveys, consensus is typically achieved when agreement or disagreement falls within the 50 to 80% range (16, 17). However, in our study, the agreement levels ranged from 80 to 100%, reflecting a high degree of alignment among the participating health care professionals. This strong agreement suggests not only a shared understanding of issues involved in managing osteoporosis in CKD patients but also a clear trend toward adopting precise approaches in clinical practice. The expert panel survey began by evaluating the question regarding the most common presentation of CKD-induced osteoporosis, with a focus on whether it is predominantly asymptomatic, detected primarily through screening, and often followed by fragility fractures. This issue is critical because early detection and management hinge on understanding the typical clinical manifestations of osteoporosis in CKD patients. More than 90% of the panelists agreed that asymptomatic presentation is indeed the most common scenario, often identified during routine screening, with bone pain and fragility fractures being less frequent but still significant (18, 19). This consensus highlights the need for proactive screening strategies to identify osteoporosis in its early stages, thereby preventing fractures and improving patient outcomes.

Identifying and managing clinical risk factors for osteoporosis and fracture risk in CKD patients is essential for effective treatment strategies. Several key risk factors have been consistently recognized, including advanced CKD stages with prolonged hemodialysis, a T-score of ≤ −2.5, older age (≥ 65 years), low BMI (≤ 20 kg/m²), postmenopausal status, extended corticosteroid therapy for post-transplant and primary glomerular diseases (>10 mg per day for more than 90 days), hyperparathyroidism, chronic malnutrition, and hyperthyroidism (6, 20, 21). These factors align with recommendations from the European consensus (22), American Society of Nephrology (6), and KDIGO guidelines (18, 19) for managing osteoporosis in CKD patients, underscoring their importance in clinical practice. During the expert panel discussion, a unanimous agreement was reached on these risk factors. However, the panelists emphasized that in addition to patients with a T-score ≤ -2.5, those with normal BMD but a history of fragility fractures should also be considered for osteoporosis treatment. This highlights the importance of a comprehensive approach to patient evaluation, where both bone density metrics and clinical history are critical in guiding management decisions.

Early screening and accurate diagnosis are crucial for implementing effective therapeutic strategies to enhance bone health in this vulnerable population. The diagnosis of osteoporosis traditionally involves evaluating bone quantity through dual energy X-ray absorptiometry (DXA) and vertebral fracture assessment (VFA), and bone quality through trabecular bone score (TBS) assessments. While general population guidelines recommend BMD screening for women ≥65 years and men ≥70 years, in CKD patients, earlier screening is advised, particularly in postmenopausal women and individuals ≥50 years, who are at a higher risk of fractures (23, 24). The panel reached a consensus on the importance of using DXA as the primary tool for osteoporosis screening in CKD patients. Furthermore, the use of fracture risk assessment tool (FRAX) for fracture risk assessment in CKD patients was discussed, noting its limitations as CKD and GFR levels are not included in the FRAX calculation. Despite this, the panel supported its use, referencing findings of Whitlock and co-workers (25), who demonstrated a stronger relationship between FRAX scores and fracture risk in CKD patients compared to those with preserved eGFR. Importantly, the World Health Organization (WHO) has clarified that the FRAX^®^ tool is not a WHO-developed or endorsed tool. WHO has no involvement in the development, validation, or evaluation of FRAX^®^ algorithms, data, or embedded treatment recommendations (26). Additionally, the panel suggested that, wherever available, combining BSAP and iPTH could be more reliable than individual measurement for distinguishing between high and low bone turnover diseases, further enhancing the precision of osteoporosis management in CKD.

The management of osteoporosis in patients with CKD, necessitating a multidisciplinary approach that incorporates both non-pharmacological life style modifications and pharmacological strategies. The selection of treatment should be tailored to the individual fracture risk, taking into account the continuum from low to very high risk. Evaluating bone turnover status is critical, as it informs the choice of therapy, ensuring that it aligns with the patient’s bone metabolism, whether it is high, low, or adynamic. For instance, in advanced CKD, or in hemodialysis patients with eGFR < 40 ml/min, bisphosphonates must be used cautiously due to the risks of adynamic bone. While randomized controlled trials (RCTs) have not shown any significant decline in eGFR with the use of oral bisphosphonates, intravenous (IV) bisphosphonates are associated with an increased risk of acute kidney injury (AKI). Therefore, IV bisphosphonates require particular caution in this patient population (13). Teriparatide, while effective, should be administered carefully in severe renal impairment due to delayed elimination and reduced efficacy in the presence of elevated PTH levels (27). Denosumab, a RANKL inhibitor, has shown to significantly increase bone mineral density and reduce fracture risk after six months of therapy, without the nephrotoxic concerns seen with other options (13, 28). In *post hoc* analysis of FREEDOM trial and its extension (29), mild-to-moderate CKD patients were evaluated for long-term safety and efficacy of denosumab. Participants across all CKD subgroups showed consistent BMD gains, low fracture rates, and comparable adverse event profiles. Safety and efficacy of denosumab remained stable in patients with CKD stages 2 and 3 over the study period. However, regular monitoring of serum calcium and PTH levels is essential to ensure safety, especially in patients with advanced CKD particularly hemodialysis patients, where the risk of hypocalcemia is higher especially if patients had not received prior vitamin D and calcium supplements (6, 13, 28). With appropriate monitoring, it remains a viable option for managing CKD-associated osteoporosis. In a line with this, the expert panel emphasized the necessity of a multidisciplinary approach in managing CKD-induced osteoporosis, however they also suggested that renal osteodystrophy (ROD) management is a prerequisite before addressing osteoporosis (6, 13). The treatment regimen should start with lifestyle modifications and ROD management, followed by specific therapies based on bone turnover status, and serum BSAP levels (13). For patients with high bone turnover, bisphosphonates or denosumab are recommended for those with eGFR >40 mL/min/1.73m^2^, while denosumab alone is suggested for those patient with eGFR <40 ml/min (6, 13). Conversely, anabolic agents are preferred for low turnover bone (6). This approach ensures that treatments are not only effective but also safe, minimizing the risk of further complications in this high-risk population. In determining the appropriate treatment approach, the choice between anti-resorptive or anabolic therapy should consider estimated fracture risk, side effect profile, local availability, and cost. **Table 3** provides detailed information on anabolic and anti-resorptive therapies, including their mechanisms of action and potential side effects.

**Table 3:** Anabolic and anti-resorptive therapies for osteoporosis in CKD patients (14).

Therapy	Mechanisms of action	Dosage	Side effects
Anti-resorptive therapy			
Bisphosphonates	Inhibits osteoclast-mediated bone resorption	Zoledronate 5 mg IV yearly Alendronate 70mg PO weekly Pamidronate 60 mcg IV per 3 months Risendronate 5mg PO daily Ibandronate 150 daily PO or 3 mg IV every 3 months	Renal toxicity; Hypocalcemia; Adynamic/atypical bone; Gastric irritation; Jaw osteonecrosis
Denosumab	RANKL inhibitor, inhibits osteoclast activity and stimulates osteoblast	60 mg subcutaneously every 6 months	Hypocalcemia; Rebound bone loss; Elevated PTH
Calcitonin	Anti-resorptive	Loading dose: 50 to 100 units of calcitonin daily (intramuscular or subcutaneous), Maintenance dose: Either 50 units daily or 50 to 100 units every 1 to 3 days Nasal spray: 200 units per actuation in alternating nostrils daily	Hypersensitivity; Risk of hypocalcemia; Risk of malignancy Nasal spray – epistaxis, rhinitis, and ulceration of the nasal mucosa
Romosozumab	Sclerostin inhibitor, increases bone formation (osteogenesis) and decreases resorption	210 mg subcutaneously monthly	Cardiovascular events; Hypocalcemia; Atypical fractures; Jaw osteonecrosis
Raloxifene	Selective estrogen receptor modulator	60 mg orally daily for a year	Venous thromboembolism; Hot flashes
Anabolic therapy			
PTH analogues (teriparatide, abaloparatide)	Stimulates bone formation by activating osteoblasts	Teriparatide: 20 µg subcutaneously daily for 2 years Abaloparatide: 80 mcg daily	Hypercalcemia; Nausea; Leg cramps; Reduces cortical bone density; Osteosarcoma in animals

Frequent monitoring is a critical aspect of managing osteoporosis in CKD patients, as it ensures that the chosen treatment strategies are both effective and safe over time. The importance of regular follow-up is well-documented/reported in numerous guidelines and recommendations, emphasizing the need for ongoing assessment to adapt therapies as needed (16, 18, 22, 23). Our expert panel concurs with these guidelines and recommendations, advocating for consistent follow-up in CKD patients with osteoporosis. At each follow-up visit, they also suggested that it is essential to monitor changes in bone mineral density (BMD) using DXA scans, as this provides a reliable measure of treatment efficacy and helps guide adjustments in therapy. DXA scans are particularly valuable in this context, offering a precise and non-invasive method to track bone density changes over time. By integrating regular DXA assessments (1–2 yearly) into the follow-up routine, clinicians can better manage osteoporosis in CKD patients, ensuring that the therapeutic approach remains aligned with the patient’s evolving needs. This approach not only optimizes bone health but also mitigates the risk of fractures, a significant concern in this vulnerable population (6, 13, 23). The panel’s recommendation aligns with the broader consensus that regular (16, 22), comprehensive monitoring is vital for effective osteoporosis management in CKD patients.

Formulating an algorithm for the diagnosis and management of osteoporosis in CKD is a promising step towards standardizing care, but it requires careful consideration and thorough analysis. In a line with this, expert panel proposed the comprehensive algorithmic flow chart that would be helpful for managing osteoporosis in CKD patients, which aligns with the algorithm of Egyptian recommendations suggest by El Miedany and co-workers (2). While patient representatives were not included in this consensus, we acknowledge their importance and plan to include their perspectives in future updates to enhance relevance and applicability.

## 5 Limitations

One of the limitation of this study is its reliance on expert opinion, which, while valuable, may introduce bias and does not replace the need for robust clinical trial data. Additionally, the lack of a Delphi method with multiple rounds of feedback could have limited the depth of consensus, potentially overlooking different opinions that may have emerged with further discussions. Further, our consensus recommendations were tailored to the Indian healthcare context, therefore we acknowledge that variations in healthcare infrastructure, resource availability, and patient demographics across different regions may affect their applicability.

## 6 Conclusion

This study provides a comprehensive expert consensus on the diagnosis and management of osteoporosis in patients with CKD. The recommendations emphasize the importance of a multidisciplinary approach, early screening, and tailored treatment strategies that consider both bone health and the underlying CKD. While further research and larger-scale studies are needed, these consensus recommendations offer a practical framework for clinicians managing osteoporosis in CKD, aiming to improve patient outcomes and address the challenges posed by this complex condition.

## Data Availability

The original contributions presented in the study are included in the article/supplementary material. Further inquiries can be directed to the corresponding author.

## Code availability

We confirm that EndNote Version 9, a freely available software application, was utilized for reference management.
